# Antiseizure Medications in Alzheimer’s Disease from Preclinical to Clinical Evidence

**DOI:** 10.3390/ijms241612639

**Published:** 2023-08-10

**Authors:** Francesca Bosco, Lorenza Guarnieri, Vincenzo Rania, Ernesto Palma, Rita Citraro, Maria Tiziana Corasaniti, Antonio Leo, Giovambattista De Sarro

**Affiliations:** 1 Department of Health Sciences, School of Medicine and Surgery, Magna Graecia University of Catanzaro, 88100 Catanzaro, Italy; fra.bosco88@gmail.com (F.B.); lorenza.guarnieri@unicz.it (L.G.); raniavincenzo1@gmail.com (V.R.); citraro@unicz.it (R.C.); desarro@unicz.it (G.D.S.); 2 Department of Health Sciences, School of Pharmacy, Magna Graecia University of Catanzaro, 88100 Catanzaro, Italy; palma@unicz.it (E.P.); mtcorasa@unicz.it (M.T.C.); 3 System and Applied Pharmacology, University Magna Graecia (FAS@UMG) Research Center, Department of Health Sciences, School of Medicine, Magna Graecia University of Catanzaro, 88100 Catanzaro, Italy

**Keywords:** Alzheimer’s disease (AD), epilepsy, epileptiform activity, seizure, antiseizure medications (ASMs)

## Abstract

Alzheimer’s disease (AD) and epilepsy are common neurological disorders in the elderly. A bi-directional link between these neurological diseases has been reported, with patients with either condition carrying almost a two-fold risk of contracting the other compared to healthy subjects. AD/epilepsy adversely affects patients’ quality of life and represents a severe public health problem. Thus, identifying the relationship between epilepsy and AD represents an ongoing challenge and continuing need. Seizures in AD patients are often unrecognized because they are often nonconvulsive and sometimes mimic some behavioral symptoms of AD. Regarding this, it has been hypothesized that epileptogenesis and neurodegeneration share common underlying mechanisms. Targeted treatment to decrease epileptiform activity could represent a valuable strategy for delaying the neurodegenerative process and related cognitive impairment. Several preclinical studies have shown that some antiseizure medications (ASMs) targeting abnormal network hyperexcitability may change the natural progression of AD. However, to date, no guidelines are available for managing seizures in AD patients because of the paucity of randomized clinical trials sufficient for answering the correlated questions. Future AD clinical studies are mandatory to update clinicians about the symptomatic treatment of seizures in AD patients and recognize whether ASM therapy could change the natural progression of the disease, thereby rescuing cognitive performance.

## 1. Epilepsy and Alzheimer’s Disease: Connection Points between Two Different Pathologies

Alzheimer’s disease (AD) represents the most common cause of dementia. In developed countries, the number of patients with AD steadily augments with increasing age [1,2]. AD occurs most frequently after the age of 65 years and is termed late-onset AD (LOAD), while it more rarely occurs before this age as early-onset AD (EOAD), which accounts for less than 5% of cases [3]. As previously reported, above all in the elderly, AD predisposes spontaneous repeated seizure (SRS) onset [4,5]. Notably, people with epilepsy (PWE) are also more likely to develop AD/dementia compared to the general population. Several studies have proposed a bi-directional link between epilepsy and AD, with patients with either disease carrying a nearly two-fold risk of contracting the other compared to healthy controls [4]. This finding should not be surprising considering that Alois Alzheimer himself, starting from his second case onwards, recognized an increased prevalence of epilepsy in AD patients [4,6,7]. Despite this, epilepsy remains an under-recognized comorbidity of Alzheimer’s disease. Possible explanations are that seizures are often nonconvulsive and overlap with some behavioral symptoms of AD, as well as the limited use of electroencephalograms (EEGs) in AD patients [8]. AD–epilepsy comorbidity negatively impacts the quality of life (QoL) of patients and their families, thus representing a serious public health problem, so identifying the relationship between seizures and AD has attracted much interest and is a very active field of research today [9]. A major goal of this research will be to clarify if epilepsy is a cause or consequence of AD. In addition, it is helpful to bear in mind that, in some diseases, cognitive impairment and seizures are symptoms linked to common pathological conditions, such as neuroinflammation, blood–brain barrier dysfunction, and neuronal loss. Several of these conditions are involved in the pathogenesis of AD and can also increase neuronal excitability, suggesting that neurodegeneration and epileptogenesis could share common underlying mechanisms [4,10,11]. It has been shown that abnormal neuronal hyperexcitability can foster cognitive decline and promote the abnormal production of neurodegenerative proteins, which consistently increases with neuronal firing. At the same time, it is well known that epileptic seizures may induce neuronal damage and neurodegeneration, accelerating cognitive impairment [12,13,14,15]. Experimental data indicated that the β-amyloid (Aβ) pro-epileptogenic effect occurs at the oligomeric stage, and its accumulation promotes neuronal hyperexcitability long before plaque deposition can be observed [12,16]. By virtue of this, a vicious cycle has been hypothesized whereby Aβ deposition leads to an excitatory-inhibitory (E/I) imbalance that promotes epileptogenesis and the related seizures onset, which in turn fosters a further deposition of neurodegenerative proteins, facilitating neuronal damage [16,17]. Although this evidence affirm3e the relationship between Aβ deposition and seizure onset, the pathogenetic mechanisms underlying this close relationship are unknown. The potential association between neurodegenerative proteins and epileptic seizures has been extensively investigated for Aβ. In contrast, limited evidence exists on the influence of α-synuclein (α-syn)-facilitated neurodegeneration in epileptogenesis [4]. While the role of α-syn in neurodegeneration is well known, it is uncertain whether hyperexcitability can be considered a fundamental element of α-synucleinopathies and Lewy body dementia (LBD) [4,16]. Clinical evidence has shown augmented expression of α-syn in PWE, underlying the possibility of α-syn-mediated neurodegeneration in epilepsy [18,19]. Likewise, preclinical data have also reported a close relationship between α-syn and epileptic seizures [20,21]. By virtue of these findings, targeting neurodegenerative proteins could represent a promising therapeutic strategy against epilepsy. Several pieces of evidence in the literature suggest that glutamate-glutamine cycle dysregulation may underpin the high risk of epilepsy in AD. However, the mechanism that links AD pathologies, such as Aβ, APP, and Tau, to alteration of the glutamate-glutamine cycle and seizure susceptibility in patients is not well clarified. Regarding this, it has been proposed that AD pathologies such as Aβ oligomers can increase the risk of seizure by disrupting the fundamental role of astrocytes in glutamate reuptake. Therefore, the disruption of glutamate-glutamine homeostasis via astrogliosis can cause neurotransmitter accumulation and the release of proinflammatory cytokines into the synaptic cleft [22]. To date, evidence in the literature supports the role of neuroinflammation in the pathogenesis of AD and epilepsy comorbidity [23]. Although several mechanisms can be relevant, studies have also uncovered that epilepsy and AD are linked strictly to ion channel activity. The modification of ion channels, mainly because of mutations in genes encoding ion channel subunits [24], can lead to the dysregulation of E/I signaling, which results in the spread of aberrant discharges in epilepsy, and a variety of ion channels, such as NMDA and AMPA glutamate receptors, are putatively involved in the pathogenesis of AD and several other neuropsychiatric conditions [25,26,27]. Interestingly, it has been shown in an experimental mouse model of cerebral amyloidosis that the D1 receptor could be involved in mediating the epileptic effects of Aβ_1–42_. This novel link between Aβ_1–42_ and D1 dopamine (DA) receptor signaling might represent a potential therapeutic target [28].

Overall, the common comorbidity of epilepsy and AD raises concerns about its cause versus its consequences. Do long-standing seizures augment the probability of contracting AD, or do neurodegenerative modifications in AD patients set the stage for seizure onset? Studies show that both mechanisms are probable, although the second scenario seems more likely [6].

While waiting to discover the precise association between neurodegeneration and epileptogenesis and answers to the questions above, targeted intervention in reducing network hyperexcitability could represent a valid therapeutic strategy to defer neurodegenerative changes and the consequent cognitive impairment in patients by several years. Since data in the literature suggest the role of seizures and epileptiform activity in cognitive dysfunction, early diagnosis and treatment of seizures in AD patients should be pursued. Various experimental studies suggest that antiseizure medications (ASMs) controlling epileptic activity could also postpone the progression of Aβ pathology and consequent dementia, representing a promising disease-modifying strategy against AD [16,29,30,31]. From the translational perspective, randomized clinical trials (RCTs) are mandatory. In fact, to date, the paucity of RCTs makes the decision on whether to treat seizures and epileptiform activity arduous in AD patients. Even more challenging is the choice of ASM for AD patients since several factors should be considered, such as the pharmacokinetic profile, comorbid illnesses, and side effects [32,33]. At odds, it is a common opinion that ASM treatment should be avoided in the absence of epileptic activity in AD patients [8].

## 2. Antiseizure Medications in Alzheimer’s Disease: Evidence from Experimental Models

Experimental models are a fundamental tool to truly recognize the pathogenic basis of epilepsy and comorbid AD and the relationship between them. Several studies in the literature have shown that different transgenic mouse models of AD have epileptic activity, also suggesting that this abnormal hyperexcitability might be a mechanism by which neurodegenerative proteins, including Aβ, produce more widespread neuronal damage [4,29,34]. Therefore, ASMs, in addition to reducing the frequency of epileptiform discharges and seizures, might also restore E/I balance and normalize synaptic function, potentially postponing the course of AD [35]. Up to now, there has been a paucity of experimental studies evaluating the impact of ASMs on disease severity in AD models. Furthermore, the doses of ASMs applied often differ among studies available in the literature.

Valproic acid (VPA), currently not indicated for AD patients because of its adverse effects on cognitive and motor functions [36], was able to counteract epileptiform activity in a dose-dependent manner in amyloid precursor protein/presenilin 1 (APP/PS1) transgenic mice. However, this effect was transitory since it disappeared 1 month after drug withdrawal [37,38]. VPA treatment (30 mg/kg, i.p.) significantly reduced neuritic plaque formation and improved learning and memory deficits in APP23 transgenic mice. Interestingly, the authors found that VPA reduced Aβ production by inhibiting glycogen synthase kinase-3β (GSK-3β)-mediated γ-secretase cleavage of APP in vivo and in vitro. These results suggested that VPA administration might be helpful in the prevention and treatment of AD [39]. In line with this study, it was previously observed that VPA could counteract the pathogenesis of AD through multiple mechanisms, including inhibition of excitotoxicity, reduction of apoptosis-activating Bcl-2, promotion of neuronal survival, and inhibition of GSK-3β, which, in addition to limiting Aβ production, also reduced Tau protein phosphorylation and the deterioration of cholinergic transmission [40]. Using cultured rat hippocampal neurons, it was also shown that VPA protected against Aβ- and glutamate-induced neurotoxicity by decreasing the increase in intracellular free calcium levels. Similar results were also observed with phenytoin (PHT) and carbamazepine (CBZ) [41]. Based on this, it was speculated that the synergistic effects between ASMs, such as VPA, and other GSK-3β inhibitors might be crucial in treating neurodegenerative diseases related to altered glutamate levels [22,39]. To date, it is well known that overactivation of glutamatergic neurotransmission in the central nervous system (CNS) is a cause of neuronal death [22,42], and this could also play a mechanistic role in the etiopathogenesis of epilepsy–AD comorbidity. As mentioned, there is a close relationship between Aβ oligomer accumulation and neuronal hyperexcitability, which could be supported by glutamate receptor dyshomeostasis [30,43]. In fact, Aβ alters the expression and trafficking of glutamate receptors, leading to Ca^2+^ dyshomeostasis and damaging synaptic plasticity with consequent long-term potentiation (LTP) suppression and long-term depression (LTD) improvement. As Aβ promotes Ca^2+^ influx, glial cells can be activated and release pro-inflammatory cytokines that decrease glutamate uptake and further weaken synaptic functions [28,43,44,45]. For these reasons, targeting glutamate receptors such as AMPAR could represent a potential therapeutic strategy to counteract seizure development and cognitive deficits in patients with AD and comorbid epilepsy [43]. Perampanel (PER), a noncompetitive α-amino-3-hydroxy-5-methyl-4-isoxazole propionate receptor (AMPAR) antagonist, has also shown neuroprotective effects in several experimental models of neurodegenerative diseases [26,30,46]. To support this hypothesis, a recent study reported that PER, without affecting physiological synaptic transmission, counteracted Aβ-induced hippocampal hyperexcitability and long-term potentiation (LTP) impairment ex vivo. PER counteracted the hippocampal-based cognitive deficits in mice injected with Aβ oligomers while maintaining antiseizure efficacy. Interestingly, the PER effects were linked to a reduction in the expression of some pro-inflammatory cytokines in this mice model [30]. Recently, a review summarizing preclinical and clinical data on AMPA antagonism supported the possibility of treating several neuropsychiatric conditions, beyond epilepsy, with PER [25].

Regarding sodium channel blockers, in male heterozygous APdE9 transgenic mice, chronic treatment with CBZ (10–40 mg/kg, t.i.d.) significantly suppressed spontaneous epileptic discharges. According to this study, 56% of mice on lower doses of CBZ and 50% on higher doses of CBZ were responders. The effectiveness of CBZ was also detected in a 3×Tg mouse model of AD. In detail, long-term CBZ treatment (100 mg/kg) significantly attenuated spatial learning and memory deficits in 3×Tg-AD mice. This improvement, induced by CBZ treatment, was associated with an increase in autophagic flux [38,47]. In an APdE9 transgenic mouse model, PHT (10–40 mg/kg, t.i.d.) decreased epileptiform activity, with responder rates comprising between 25% and 80%, although side effects were detected. The efficacy of PHT was not reported in other studies; in fact, this ASM had a slight impact on SWDs in either APP/PS1 or 3×Tg mice [38,48]. Aggravation of SWDs and cognitive dysfunction due to PHT have also been detected in human amyloid precursor protein (hAPP) transgenic mice [49,50].

Zonisamide (ZNS) acts through different mechanisms, such as modulation of voltage-gated ion channels, reducing extracellular GABA levels, and preventing caspase-3 activation. Recently, it was shown that ZNS (40 mg/kg/daily, for 16 weeks) could improve cognitive impairment by enhancing PSD95 and CREB expression in type 2 diabetes mellitus (T2DM) mice, diminish the Aβ load, and rescue Tau hyperphosphorylation by decreasing the activity of JNK. According to the authors, ZNS could be a new compound for managing dementia in T2DM [51]. Despite this, ZNS has been associated with adverse cognitive and mood effects in humans [52]. Interestingly, it was found that rufinamide (RUF; 3 mg/kg, for 4 weeks) administration counteracted learning and memory deficits and increased neurogenesis in the dentate gyrus of older adult gerbils, enhancing expression of IGF-1, IGF-1R, and p-CREB [53]. To date, evidence for clinical trials shows that RUF seems to have a favorable cognitive effect profile in patients with Lennox-Gastaut syndrome [54].

Lacosamide (LCS) and lamotrigine (LTG), other sodium channel blockers, have been associated with positive effects in AD preclinical models. Notably, chronic treatment with LTG (30 mg/kg, daily), a broad-spectrum ASM, reduced learning and memory impairment in APP/PS1 transgenic mice. Such effects of LTG were linked to its ability to suppress abnormal cortical hyperexcitability, enhance levels of neurotrophic factors, and decrease Aβ generation and deposition in this mouse model [55]. In addition, a very recent study on Tg 2576 mice also showed that LTG (10 mg/kg) could reduce seizure-induced cognitive deficits in the early stages of AD [56]. A study performed in APP/PS1 mice reported that LTG improved cognitive-like deficits by reducing synapse and neuronal damage in the CNS. Interestingly, high-throughput RNA sequencing showed that the neuroprotective effects of LTG in this transgenic mouse model could be achieved by modulating the brain expression of several markers involved in neuroinflammation, Aβ production, and Tau hyperphosphorylation, such as *Ptgds*, *Cd74*, *Map3k1*, *Fosb*, and *Spp1* [57].

LCS has been reported to have neuroprotective effects and histone deacetylase inhibition activity in several preclinical models [58]. Recently, chronic LCS treatment (30 mg/kg, daily) decreased streptozotocin (STZ)-induced cognitive deficits in male Wistar rats. Such effects of LCS were correlated with a reduction in Aβ and Tau protein formation [59]. Similarly, the pharmacological effects of levetiracetam (LEV) have been tested on cognitive impairment related to the focal injection of STZ, as a model of AD, in rats. The authors also evaluated the protective effects of LEV against hippocampal cell loss, oxidative stress, acetylcholinesterase (AChE) enzyme activity, neuroinflammation, and Tau protein deposition. From this study, it emerged that LEV (100 and 150 mg/kg) reduced neuronal death and cognitive-like decline in STZ-induced AD by suppressing hippocampal neuronal loss, restoring changes in redox status, rebalancing acetylcholinesterase activity, and suppressing the expression of proinflammatory cytokines and the hyperphosphorylation of Tau [60]. Likewise, Sanchez et al. observed that only LEV (5, 50, or 200 mg/kg), among the ASMs studied, significantly reduced abnormal spike activity, in a dose-dependent manner, in hAPP transgenic mice. Ethosuximide (ETH), gabapentin (GAB), PHT, VPA, and vigabatrin (VGB) did not significantly modify spike frequency in this mouse model. Pregabalin (PGB) exacerbated electrographic spike frequency in hAPPJ20 mice. Furthermore, chronic treatment with LEV was able to reverse hippocampal remodeling and decrease behavioral abnormalities, synaptic dysfunction, and learning and memory impairment in this mouse model of AD [49]. Similarly, LEV (75 mg/kg/i.p. administered 3 times per day for 2 weeks) restored neurogenesis and improved performance in a neurogenesis-associated spatial discrimination task in a transgenic APP mouse model of AD [61].

Brivaracetam (BRV; 8.5 mg/kg/day), an AMS closely related to LEV, and ethosuximide (30 mg/mL) reduced spike-wave discharges (SWDs) detected in two well-validated transgenic mouse models of AD (APP/PS1 and 3×Tg); however, only BRV was able to reverse spatial memory deficits in mice [48]. This data could support the potential role of SV2A protein in epilepsy–AD comorbidity. Regarding this, it has also been shown that a subcutaneous treatment with BRV (10 mg/kg) and LEV (150 mg/kg) for 28 days, before and during amygdala kindling, significantly delayed the progression of seizure severity in aged Tg2576 mice. According to the authors, targeting SV2A could be a valuable therapeutic strategy for preventing epilepsy in AD patients [62].

Topiramate (TPM; 20 mg/kg, for 30 days) and LEV (50 mg/kg, for 30 days) improved behavioral impairment and reduced Aβ plaques in APP/PS1 transgenic mice. In detail, the results showed that TPM and LEV augmented Aβ clearance and upregulated Aβ transport across the blood–brain barrier and autophagic digestion. These ASMs were also able to normalize the activation of AMPK/Akt/GSK3β in vivo and in vitro. TPM and LEV also inhibited histone deacetylase activity in transgenic mice [63]. In 2014, using a rat model of amyloidosis, it was also shown that TPM counteracted apoptosis, enhancing the expression of Bcl-2 and reducing the expression of Fas, Bax, and Caspase-3 in hippocampal neurons. This evidence provided insight into the protective effect of TPM against neuronal death [64]. Regarding TPM, Owona et al. also reported that TPM (20 mg/kg, for 21 days) restored the frequency of interactive behavior and nest-building activity, possibly both diminishing the deposition and aggregation of Aβ and the activation of microglia in the cortex and hippocampus of APP/PS1 transgenic mice [65]. Despite these promising preclinical studies, RCTs indicated that TPM was linked to adverse effects on cognition in PWE and healthy volunteers [66,67,68].

Cannabidiol (CBD), a phytocannabinoid devoid of psychoactive responses, has been approved for the treatment of some forms of refractory epilepsy; to date, there is also great interest in its off-label use [69,70,71]. Preclinical studies have proposed that CBD can alleviate cognitive impairment, Aβ-induced neuroinflammation, oxidative responses, and neuronal death [72]. CBD (2.5 or 10 mg/kg, for 7 days) was also able to reduce, in a dose-dependent manner, the expression of glial fibrillary acid protein (GFAP), a marker of astroglial activation, in mice that underwent intrahippocampal injection of human Aβ. In addition, CBD also decreased iNOS and interleukin-1β expression, attenuating Aβ-induced pro-inflammatory responses in this model [73]. Likewise, it was observed that chronic CBD treatment, at 20 mg/kg, was able to rescue spatial learning deficits both in Aβ-injected and APP/PS1 transgenic mice. Such effects of CBD were related to its ability to counteract Aβ-mediated neuroinflammation [74,75]. The CBD preventive effect on AD onset was also studied in APP/PS1 transgenic mice. To this aim, vehicle and AD transgenic mice were treated orally with CBD at 20 mg/kg daily for 8 months. Interestingly, this study proved CBD’s ability to counteract the onset of social recognition impairment in these mice [76]. The impaired social recognition and spatial reversal learning in APP/PS1 transgenic mice were also restored after intraperitoneal treatment with CBD at 50 mg/kg for 3 weeks. The authors observed that CBD treatment moderately decreased insoluble Aβ deposition in the hippocampus of 12-month-old transgenic mice. At odds, it did not affect neuroinflammation, neurodegeneration, or the expression of PPARγ markers in the cortex [77]. These findings emphasized the potential role of CBD as a valuable therapeutic strategy for AD patients. Notably, similar effects were also shown for the acidic variants of CBD and THC. In particular, cannabidiolic acid (CBDA) and tetrahydrocannabinolic acid (THCA) improved cognitive decline by modulating Ca^2+^ levels and hippocampal pathology in a mouse model of amyloidosis (mice injected with Aβ into the hippocampus) [78] (Table 1).

Interestingly, for the first time, it was shown that young, female transgenic AD mice with increased soluble Aβ_1–42_ levels (APP/PS1 mice) or both increased soluble Aβ_1–42_ and t-Tau (3×Tg mice) brain levels were more susceptible to 6 Hz kindled seizures and were more rapidly kindled. The anticonvulsive effects of BRV, LEV, and LTG were less pronounced in the fully kindled AD mice, pointing to pharmacoresistance in both AD mouse strains and suggesting that seizures of young AD mice are more difficult to treat [79].

Overall, the currently available studies have mainly been performed in transgenic animal models, which, despite providing much information on AD pathogenesis, limits their validity due to realities such as most AD mouse models not developing substantial synaptic and neuronal loss, as detected in the brain of AD patients. Several of these studies were mainly performed in mice overexpressing human genes linked to familial AD (FAD), resulting in the formation of Aβ plaques. However, AD is characterized by the presence and interplay of both Aβ formation and neurofibrillary tangle pathology. These models essentially mimicked early-onset AD and provided no information about how sporadic forms develop [80,81,82]. It seems likely that AD progression is affected by two main factors: genetic risk factors and risk factors associated with lifestyle, possibly related to vascular risk factors. Other limitations of these transgenic models are linked to the smaller and less-developed prefrontal cortex and shorter lifespan of the animals, which makes them unsuitable for studying age-related neurodegenerative diseases such as AD. Notably, there are also considerable differences between rodents and human immune systems [83]. Therefore, none of the current animal models can replicate the complexity of the disease as observed in human patients. In fact, it has been reported that models reflecting only single features of AD pathogenesis do not mimic AD. Therefore, new experimental models that incorporate genetics with environmental interactions, timing of disease development, heterogeneous mechanisms and pathways, comorbidities, and other pathologies that lead to AD are mandatory.

## 3. Antiseizure Medications in Alzheimer’s Disease: Evidence from Clinical Trials

**Figure 1:**
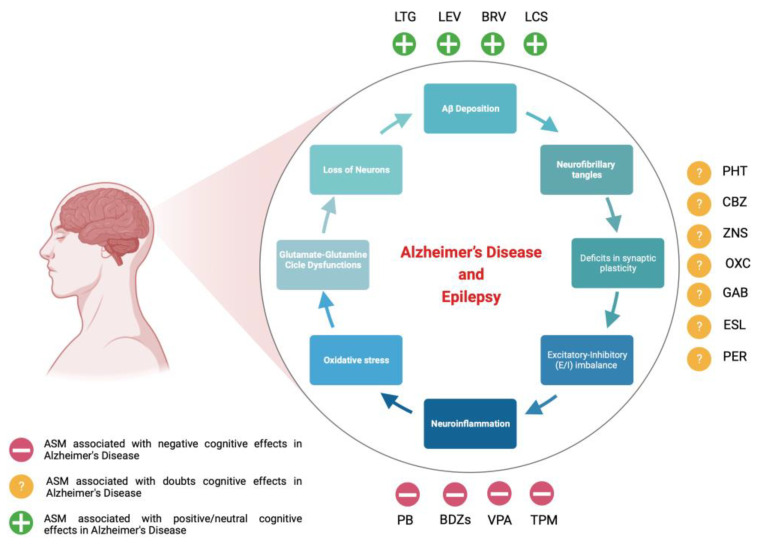
Diagram depicting the main molecular pathways involved in Alzheimer’s disease–epilepsy comorbidity and ASM effects on cognitive trajectory in Alzheimer’s disease (adapted from [94]).

Since various pieces of evidence support the detrimental role of subclinical epileptiform discharge and/or seizures on cognitive trajectory, early diagnosis and treatment of epileptic activity in AD patients should be pursued. ASMs could be expected to have beneficial effects on cognition by regulating neuronal networks and Aβ accumulation. Despite the medical armamentarium against seizures having been enriched with newer ASMs, up to now, the decision to treat seizures and the choice of ASM for AD patients has been considered a complex clinical challenge. Even more difficult is the decision to treat subclinical epileptiform activity [4,6]. Although preclinical studies have highlighted the effectiveness of ASMs in AD, to date, only a few RCTs have been performed to evaluate the efficacy and safety of ASMs in AD patients. These clinical studies are often ambiguous because of experimental design limitations, especially the insufficient number of patients [4,84]. Therefore, the choice of ASM by clinicians should be based on data acquired from RCTs investigating the pharmacological profile of ASMs in elderly subjects with or without AD [85]. Before starting treatment, it is desirable to bear in mind that ASM selection for the elderly needs more care than in younger patients for various reasons, including age-related decline in liver and kidney functions and levels of plasma-binding proteins, thus making these patients a difficult group to treat. This is further made challenging by the presence of comorbidities and polytherapies that augment the possibility of drug interactions. Moreover, ASMs are often associated with adverse effects (AEs) that could be tolerated in younger subjects but can be devastating for the elderly [86,87,88]. Sometimes, ASMs (notably, PHT and VPA) can have proconvulsant effects, particularly at high doses [89,90]. Drugs generally used in the treatment of AD patients, including acetylcholinesterase inhibitors, noncompetitive N-methyl-D-aspartate receptor (NMDA) antagonists, antidepressants, and neuroleptics, can reduce the seizure threshold [85,91,92]. Basically, it has been reported that the cognitive adverse events (CAEs) of ASMs are mainly associated with first-generation drugs; on the contrary, newer ASMs, including GAB, LEV, BRV, LTG, LAC, and PER, have shown a favorable safety profile on cognitive function (Figure 1) [25,32,93].

Interestingly, several RCTs performed on geriatric patients with new-onset seizures depicted that CAEs were less marked in patients taking GAB, LTG, or LEV compared to patients taking CBZ. Seizure control did not differ among groups in these studies. Accordingly, these ASMs should be used as the initial treatment for newly diagnosed epilepsy in the elderly [95,96,97].

This evidence was further supported by a retrospective, uncontrolled trial performed from 2000 to 2005 to compare the effectiveness and tolerability of 10 ASMs (i.e., LTG, GAB, LEV, CBZ, PHT, and TPM) in 417 PWE aged 55 years or older. The percentage of patients who remained taking the ASM for 12 or more months (12-month “retention”) was also evaluated. Overall, 329 patients continued treatment with an ASMs for 1 year, showing a 12-month retention rate of 65%. LTG showed the highest retention rate (78.6%) along with LEV (72.5%), compared to CBZ (48.4%), GAB (59%), PHT (59.3%), and TPM (55.6), while OXC showed the lowest retention rate (23.5%) compared to all other ASMs. At odds, in the subgroup of patients with refractory epilepsy, VPA showed a remarkably high retention rate (90%) together with LTG (77.8%), whereas LEV, ZNS, and TPM showed a slight retention rate (approximately 70%). Conversely, in terms of seizure freedom, LTG exhibited the highest 12-month seizure freedom rate (54.1%), followed by LEV (46.2%), GAB (18.5%), and OXC (9.5%). Similar results were reported in the subgroup of patients with refractory epilepsy; particularly, the highest rates of seizure freedom were achieved with LTG (47.4%) and LEV (38.9%). Both of these rates were significantly higher than those of the other ASMs. Finally, it was observed that 29.1% of patients enrolled in this study experienced an intolerable AE. Imbalance, drowsiness, and gastrointestinal (GI) symptoms were the most common intolerable AEs. Particularly, LTG caused significant imbalance (13.8%) compared to GAB, LEV, PHT, OXC, and VPA, along with drowsiness (8.8%), GI disorders (8.8%), dizziness (6.8%), CAEs (5.4%), allergies (4.9%), and psychiatric AEs (4.3%). Drowsiness occurred mainly with CBZ, GAB, LEV, OXC, and TPM (>10% of patients required lowering of dose). Dizziness was another common intolerable AE ascribed mainly to GAB, LTG, OXC, PHT, and ZNS. The most common AEs for LEV were drowsiness (23.3%) and psychiatric AEs (14.2%). PHT intolerability was most often because of allergies (9.1%); TPM intolerability was because of CAEs (24%) and drowsiness (20%); VPA intolerability was because of tremors (10.8%); and ZNS intolerability was because of CAEs and GI disorders (13.6% each). According to the authors, LTG and LEV were more effective compared to other ASMs. Moreover, LTG and LEV did not appear to negatively affect cognitive function, thus supporting their use in the elderly [98]. Based on this data, ASMs, including CBZ and OXC, that have shown doubtful effects on cognition should be avoided in AD patients. The same conclusion can be drawn for VPA; in fact, in placebo-controlled RCTs, VPA administration was not able to delay cognitive decline in patients with AD and it was associated with severe side effects. Notably, in two RCTs, during the first 12 months of follow-up, VPA (10–12 mg/kg/day)-treated AD patients showed an increased decline in mini-mental state exam (MMSE) scores and significant brain volume loss compared to the placebo group [99,100,101].

Likewise, phenobarbital (PB), benzodiazepines (BDZs), and TPM were associated with an increased risk of eliciting cognitive deficits, so these ASMs should be forbidden for AD patients with or without seizures; a similar conclusion can be drawn for ZNS, the effects of which on cognitive function need to be clarified [67,91,102,103,104]. Regarding PB, its detrimental cognitive effects were also documented in a prospective, randomized, three-arm, parallel-group, case–control study enrolling 95 patients (41 males, 54 females; mean age of 71.75 years) with AD–seizures comorbidity, taking LEV (n = 38), PB (n = 28), and LTG (n = 29). All patients received concomitant cholinesterase inhibitor therapy for AD. These three groups were compared to a control group (n = 68) to detect the cognitive effects of the ASMs. The pharmacological effects of these ASMs were evaluated both at baseline and after 6 and 12 months of treatment. According to the authors, there were no significant differences in seizure freedom among these ASMs. However, LEV treatment (range = 500–2000 mg/day, mean daily dose = 955.9 mg/day) was linked to improved cognitive performance (mini-mental state exam scores evaluated at the end of the observation period mirrored improvement by a mean of + 0.23 points), making it a cognitively safe compound for managing seizures in AD patients. At odds, PB-treated patients (range = 50–100 mg/day, mean daily dose = 90 mg/day) experienced a worsening of existing cognitive impairment at both 6 and 12 months post-randomization based on the mini-mental state exam (MMSE) and Alzheimer’s disease assessment scale–cognitive subscale (ADAS–Cog). During the 12-month follow-up, LTG-treated patients (range = 25–100 mg/day, mean daily dose = 57.5 mg/day) did not have a significant decline in MMSE scores. However, LTG positively influenced mood, thus making it a good choice for managing AD-associated neuropsychiatric symptoms. Treatment-emergent AEs occurred more frequently in PB-treated patients; 17% of PB-treated patients discontinued treatment. The most frequently reported AEs for PB were somnolence and asthenia. No patients withdrew from LEV and LTG treatment because of side effects. At odds, LEV had a benign neuropsychological side effect profile, making it a cognitively safe ASM for managing established seizures in older patients with AD (although there are some limitations to this observation, such as the small number of patients studied and choice of comparator) [105]. Regarding PHT, there are conflicting data in clinical settings; although cognitive deficits have been reported with PHT [6,106], it is sometimes prescribed in the elderly and AD patients [4]. The pharmacological rationale for prescribing this ASM in older PWE with cognitive decline appears to lie in a retrospective study [107]. As reported, until now, few RCTs have investigated whether an ASM can provide beneficial effects and good tolerability in AD patients (Table 2).

A Cochrane systemic review, including one RCT on pharmacological interventions in 95 AD patients, was conducted to assess the beneficial effects and safety of ASMs in AD patients. Overall, this systemic review did not offer satisfactory results to support the use of LEV, PB, or LTG in managing seizures in AD patients. No significant differences in efficacy and tolerability were reported among these ASMs. Regarding the proportion of participants with seizure freedom at 12 months, no significant differences were observed for the comparisons of LEV vs. LTG (RR 1.20, 95% CI 0.53 to 2.71), LEV vs. PB (RR 1.01, 95% CI 0.47 to 2.19), or LTG vs. PB (RR 0.84, 95% CI 0.35 to 2.02). It appeared that LEV could improve cognition and LTG could alleviate depression, while PB and LTG could aggravate cognitive impairment, and LEV and PB could exacerbate mood disorders. The authors concluded that large RCTs with a double-blinded, parallel-group design are required to corroborate the beneficial effects and good tolerability of ASMs in AD patients [108].

**Table 2:** Randomized clinical trials (RCTs) of antiseizure medications (ASMs) in Alzheimer’s disease (AD).

ASMs	Dose/Duration	Study Type/Patients	Clinical Benefits	Side Effects	References
VPA	10–12 mg/kg/day.	Randomized, double-blinded, placebo-controlled; 313 patients with mild-to-moderate AD. Study performed from 1 November 2005 to 31 March 2009.	No clinical benefits were reported.	Worsening of existing cognitive impairment and greater brain atrophy.	[99]
VPA	10–12 mg/kg/day.	Randomized, double-blinded, placebo- controlled (24 months); 313 patients with moderate AD.	No clinical benefits were reported.	Worsening of existing cognitive impairment and increased hippocampal volume loss.	[101]
LTG	25–100 mg/day; 1-year evaluation period.	Randomized, three parallel treatment groups; patients with AD–epilepsy comorbidity.	Improved score in the Cornell depression scale, with 59% responder rate at 1 year.	Seven patients described mild AEs: somnolence, dizziness, and headache. Slight decreases in MMSE and ADAS–Cog scores. No patients stopped treatment because of AEs.	[105]
LEV	1000–1500 mg/day; 1-year evaluation period.	Randomized, three parallel treatment groups; patients with AD–epilepsy comorbidity.	Improved MMSE and ADAS–Cog scores; 29% became seizure-free, 71% responder rate at 1 year.	AEs reported: dizziness, headache, asthenia, and somnolence. No patients stopped treatment because of AEs.	[105]
PB	25–100 mg/day; 1-year evaluation period	Randomized, three parallel treatment groups; patients with AD–epilepsy comorbidity.	No significant differences among LEV, LTG, and PB in seizure freedom, 64% responder rate after 1 year.	Worsening of existing cognitive impairment. Twelve patients reported AEs: somnolence and asthenia. Seventeen patients reported side effects. Five patients stopped treatment because of side effects.	[105]
LEV	125 mg/day twice daily, for 1 month.	Phase 2, randomized, double-blinded, placebo-controlled clinical study; 17 LEV-treated patients and 17 placebo-treated patients	Thirteen AD patients without epileptiform activity did not improve executive functions, whereas nine AD patients with epileptiform activity showed a significant improvement in spatial memory and executive function. LEV vs. placebo, no significant differences in cognitive function	AEs reported: dizziness, headache, vivid dreams, and gastrointestinal discomfort	[109]

Abbreviations: Adverse events (AEs); Alzheimer’s disease (AD); Alzheimer’s disease assessment scale–cognitive subscale (ADAS–Cog); Antiseizure medications (ASMs); Lamotrigine (LTG); Levetiracetam (LEV); Mini-mental state exam (MMSE); Phenobarbital (PB); Randomized clinical trials (RCTs); Valproic acid (VPA).

Notably, a phase 2, double-blinded, placebo-controlled, crossover clinical trial of 34 adult AD patients was performed between October 2014 and July 2020 to evaluate the effectiveness and tolerability of LEV (125 mg, twice daily for 4 weeks) in AD patients with and without epileptiform activity. In detail, the primary outcome of this study was to investigate the ability of LEV to improve executive function in AD patients without epileptiform activity, whereas the second outcome was cognition and disability. Of the 34 patients (mean [SD] age, 62.3 [7.7] years; 21 women [61.8%] and 13 men [38.2%]) enrolled in the study, 33 had biomarkers that were consistent with AD diagnosis; 1 patient with biomarkers that were inconsistent with AD diagnosis was excluded from the study. Overall, 5 patients (14.7%) withdrew from intervention, and 28 patients (82.4%) finished the study, 10 of whom (35.7%) displayed epileptiform activity. In this RCT, LEV, which was well tolerated, did not improve executive function in AD patients without epileptiform activity. At odds, LEV was able to recover executive function and spatial memory in AD patients with seizures or epileptiform discharges. According to the authors, this data could lead to tailored approaches to AD, in which patients with the epileptic variant of AD would receive distinctive treatments from patients without the epileptic variant. This study had several limitations, including small sample size, a population with early-onset AD and consequent selection bias, and infrequent epileptiform activity, making it difficult to quantify the pharmacological effects of LEV [109]. A recent study retrospectively compared 19 AD patients with epilepsy treated with ASMs against 16 non-epileptic AD patients, in terms of seizure response, tolerability, and cognitive performance. At baseline, AD patients with epilepsy had more cognitive fluctuation than those without epilepsy. Interestingly, during the follow-up period, seizures were well controlled with ASMs in epileptic AD patients. During this period, the epileptic AD patients showed similar cognitive performance to the non-epileptic AD patients. Therefore, seizures could represent an ASM-modifiable cognitive worsening factor of AD [110].

## 4. Conclusions

The relationship between epilepsy and cognitive impairment is poorly understood, with uncertainties regarding the mechanisms underpinning epileptic activity development in AD patients and whether epilepsy drives dementia or vice versa. For this purpose, preclinical models can undoubtedly represent, despite some limitations, a remarkable tool to delineate the links between AD and epilepsy. The currently available experimental models of AD have provided helpful information concerning the pathophysiological processes underpinning this neurodegenerative disease. Now, it is fundamental to expand the application of these models to comorbid conditions such as epilepsy and mood disorders to improve clinical management and further clarify the mechanisms of hyperexcitability and its impact on AD trajectory. Performing the forthcoming studies of seizures in different AD models will better inform future ASM discoveries and their application in older patients. Unfortunately, few studies have been performed to investigate ASM efficacy in pre-clinical models of late-onset epilepsy. Older adults are more vulnerable to seizures and epilepsy but less resistant to ASMs than young people with epilepsy. Experimental studies on aged animals and studies exploring the effect of advanced age on epilepsy and seizures are limited [111]. Detecting the potential therapeutic effect of ASMs in aging-related disorder models with the added consideration of polypharmacy is also needed.

Similarly, the benefits of using ASMs in AD patients in a clinical setting are still uncertain, mainly because of the paucity of RCTs mandatory to address this query. Treating seizures in AD patients requires a careful balance between managing seizures and treating cognitive disorders. In AD patients with neuropsychiatric symptoms, the choice of concomitant pharmacological therapy with neuroleptics, benzodiazepines, and antidepressants, which influence the seizure threshold, should also be carefully estimated. Third-generation ASMs are more suitable for treating elderly patients with epilepsy and neurodegenerative diseases because of their favorable pharmacokinetic properties and advantages in tolerability and safety. Despite this, RCTs are both qualitatively and quantitatively limited, and, until now, inconsistent. Further well-performed clinical trials are undoubtedly required to evaluate the therapeutic profile of ASMs in AD patients, although the currently available evidence seems promising. To this aim, large, prospective, double-blinded RCTs are needed not only to update clinicians about the symptomatic treatment of seizures in AD patients but also to recognize whether ASM therapy could modify the natural progression of the disease, thereby rescuing cognitive performance and postponing decline. This latter aspect deserves to be evaluated in AD patients with and without epileptic activity. Similarly, future studies should also be directed toward detecting AD patients with subclinical epileptiform activity, non-convulsive seizures, or larger populations that could benefit from network-stabilizing approaches; video EEG can provide a beneficial non-invasive biomarker to establish who can benefit from specific treatments among AD patients. Since the usefulness of ASMs lies in neuronal network stabilization, regardless of the underlying cause of the network alteration, ASMs may also be beneficial to other types of dementia beyond AD. If an ASM was proven to positively impact cognitive trajectory, this would be groundbreaking.

## Figures and Tables

**Table 1:** Antiseizure medications (ASMs) in Alzheimer’s disease (AD) models.

ASMs	Dose/Duration	Preclinical Model	Benefits	Mechanisms of Action	References
VPA	VPA daily injected at 30 mg/kg, for 1 week. After a 3-week wash-out, the same animals received injections of a higher dose of VPA at 300 mg/kg.	Male APdE9 mice (15 weeks old) and APP/PS1 transgenic mice.	Reduced epileptiform discharges for at least 1 week after treatment discontinuation, but there were no consistent long-term effects on epileptiform activity after treatment withdrawal.	Increased Histone3, Histone4, and H4K12 acetylation at promoters of genes implicated in memory formation and synaptic plasticity.	[37]
VPA	VPA daily injected at 30 mg/kg, for 4 weeks.	APP23 transgenic mice (6–7 weeks old).	Improved learning and memory deficits; reduced Aβ production.	Inhibited GSK-3β-mediated γ-secretase cleavage of APP in vivo and in vitro.	[39]
PHT; CBZ and VPA	PHT: 100 nM–1 μM, for 6–10 days; CBZ: 100 nM–10 μM, for 6–10 days; VPA: 100 nM–100 μM for, 6–10 days.	Cultured rat hippocampal neurons.	Protected against Aβ- and glutamate-induced neurotoxicity.	Reduced intracellular free calcium levels and Tau protein levels.	[41]
PER	Dentate gyrus granule cells in control condition and in presence of 0.1 nM PER. PER at 1 mg/kg/os in mice.	Aβ_1–42_-induced neurotoxicity in vitro. C57BL/6 male mice injected with Aβ_1–42_ into the right dorsal hippocampus.	Counteracted Aβ-induced hippocampal LTP impairment and hippocampal-based cognitive deficits in Aβ oligomer-injected mice while retaining antiseizure efficacy.	AMPAR antagonism. Reduced pro-inflammatory cytokine levels.	[30]
PER	PER daily injected at 1 and 5 mg/kg, for 7 weeks.	Rat transient middle cerebral artery occlusion model.	Improved spatial working memory.	Inhibited microglial activation, pro-inflammatory cytokine expression, and oxidative stress. Downregulation of Bcl-2 with activation of Akt.	[46]
CBZ	CBZ orally administered at 100 mg/kg, for 2 months.	3×Tg mouse model of AD (6 months old).	Decreased spatial learning and memory deficits in 3×Tg-AD mice.	Increased autophagic flux.	[47]
PHT	PHT daily injected at 10–40 mg/kg (t.i.d.), for 21 days.	APdE9 transgenic mouse model.	Decreased epileptiform activity, with responder rates comprising between 25% and 80%, although side effects were detected.	Blocking of sodium channels.	[38]
PHT	PHT daily injected at 10–40 mg/kg (t.i.d.), for 42 days.	APP/PS1 or 3×Tg old mice.	Reduced the number of spontaneous electrographic epileptiform discharges.	Blocking of sodium channels.	[38]
ZNS	ZNS intragastrically administrated at 40 mg/kg/, for 16 weeks.	Mouse model of type 2 diabetes mellitus with high-fat diet/STZ-induced C57BL/6J mice (4 weeks old)	Improved cognitive impairment.	Enhanced PSD95 and CREB expression in type 2 diabetes mellitus mice, diminished Aβ load, and rescued Tau hyperphosphorylation by decreasing the activity of JNK.	[51]
RUF	RUF daily injected at 3 mg/kg, for 4 weeks.	Aged gerbils (24 months old)	Reduced learning and memory deficits.	Increased neurogenesis in the dentate gyrus, and enhanced expression of IGF-1, IGF-1R, and p-CREB.	[53]
LTG	Mice received standard laboratory chow supplemented with LTG at 30 mg/kg, for 6–8 months.	APP/PS1 transgenic mice (3 months old)	Reduced learning and memory impairment.	Suppressed abnormal cortical hyperexcitability, enhanced levels of neurotrophic factors, and decreased Aβ generation and deposition in this mouse model.	[55]
LTG	LTG daily injected at 10 mg/kg, for 23 days.	A transgenic model of pre-symptomatic AD Tg2576 mice (1–2 months old)	Restored electrophysiological alterations, prevented memory deficits, and increased extracellular Aβ levels.	Restored neuronal excitability, prevented aberrant modulation of extracellular Aβ, and reduced β-secretase cleavage of APP.	[56]
LTG	LTG intragastrically administrated at 30 mg/kg, for 3 months.	APP/PS1 mice (5 months old)	Ameliorated cognitive-like deficits, reducing synapse and neuronal damage in the CNS.	Modulated the brain expression of several markers involved in neuroinflammation, Aβ production, and Tau hyperphosphorylation, such as Ptgds, Cd74, Map3k1, Fosb, and Spp1.	[57]
LCS	LCS injected at 30 mg/kg, for 21 days.	Wistar rats	Decreased streptozotocin-induced cognitive deficits.	Decreased Aβ and Tau protein formation.	[59]
LEV	LEV i.c.v. administered at 100 and 150 mg/kg, for 28 days.	Wistar rats weighing 200 ± 20 g	Reduced neuronal death and cognitive-like decline in STZ- induced AD.	Suppressed STZ-induced hippocampal neuronal loss, restored changes in redox status, rebalanced acetylcholinesterase activity, and suppressed expression of proinflammatory cytokines and hyperphosphorylation of Tau.	[60]
LEV	LEV intraperitoneal injected at 5, 50, or 200 mg/kg, for 8 days.	hAPP transgenic mice	Reduced abnormal spike activity, in a dose-dependent manner	Reversed hippocampal remodeling and decreased behavioral abnormalities, synaptic dysfunction, and learning and memory impairment in this mouse model of AD.	[49]
LEV	LEV, 75 mg/kg/i.p. administered 3 times per day, for 2 weeks.	hAPP transgenic mouse model	Improved performance in a neurogenesis-associated spatial discrimination task	Restored neurogenesis.	[48]
BRV ETH	BRV injected at 8.5 mg/kg/day, for 28 days; ETH delivered in drinking water at a concentration of 30 mg/kg, for 28 days.	APP/PS1 and 3×Tg-AD Mouse models	Reduced spike-wave discharges (SWDs) were detected. Only BRV was able to reverse spatial memory deficits in mice	Brivaracetam interacted with SV2A.	[48]
BRV and LEV	BRV subcutaneously injected at 10 mg/kg and LEV subcutaneously injected at 150 mg/kg, for 28 days.	Aged Tg2576 mice (13–25 months old)	Delayed the progression of seizure severity	Targeting SV2A	[62]
TPM and LEV	TPM intraperitoneally injected at 20 mg/kg and LEV intraperitoneally injected at 50 mg/kg, for 30 days.	APP/PS1 transgenic mice (9–7 months old)	Improved behavioral impairment and reduced Aβ plaques activity in transgenic mice	TPM and LEV augmented Aβ clearance and u-regulated Aβ transport across the blood–brain barrier and autophagic digestion. Normalized the activation of AMPK/Akt/GSK3β in vivo and in vitro. TPM and LEV were also able to inhibit histone deacetylase.	[63]
TPM	TPM intraperitoneally injected at 20 mg/kg, for 30 days.	Adult Wistar rats.	Counteracted apoptosis.	Enhanced expression of Bcl-2 and reduced expression of Fas, Bax, and Caspase-3 in hippocampal neurons.	[64]
TPM	TPM administered at 20 mg/kg, for 21 days.	APP/PS1 transgenic mice (5 months old).	Restored the frequency of interactive behavior and nest-building activity.	Diminished deposition and aggregation of Aβ. Reduced activation of microglia in the cortex and hippocampus.	[65]
CBD	CBD intraperitoneally injected at 2.5 or 10 mg/kg, for 7 days.	C57BL/6J mice (3–5 months old) subjected to administration of 10 ng of Aβ (1–42).	Modulated neuronal damage.	Reduced glial fibrillary acid protein (GFAP) expression in a dose-dependent manner. Decreased iNOS and interleukin-1β expression, thus attenuating Aβ-induced pro-inflammatory responses.	[73]
CBD	CBD intraperitoneally injected at 20 mg/kg, for 3 weeks.	Aβ-injected and APP/PS1 transgenic mice (3 months old).	Rescued spatial learning deficits.	Counteracted Aβ-mediated neuroinflammation.	[74,75]
CBD	CBD administered at 20 mg/kg, for 8 months.	APP/PS1 transgenic mice (10 weeks old).	Reduced the onset of social recognition impairment in mice.	Reduced neuroinflammation.	[76]
CBD	CBD administered at 50 mg/kg, for 3 weeks.	AβPPxPS1 transgenic mouse model (10 weeks old).	Reversed social and object recognition memory deficits.	Decreased insoluble Aβ deposition in the hippocampus of 12-month-old transgenic mice. No effects on neuroinflammation, neurodegeneration, or PPARγ markers in the cortex.	[77]
CBDA and THCA	CBDA (6 μmol/mouse) or THCA (12 μmol/mouse) injected into the hippocampus of mice.	Aβ_1–42_-injected mouse model (intrahippocampal injection).	Improved learning and memory decline in Aβ_1–42_-injected mice.	Decreased Aβ aggregation and p-Tau pathology in the hippocampus of Aβ_1–42_-injected mice.	[78]

Abbreviations: Alzheimer’s disease (AD); Amyloid precursor protein/presenilin 1 (APP/PS1); Amyloid β (Aβ); AMPA receptors (AMPARs); Brivaracetam (BRV); Cannabidiol (CBD); Cannabidiolic acid (CBDA); Carbamazepine (CBZ); Ethosuximide (ETH); Glycogen synthase kinase-3β (GSK-3β); Lamotrigine (LTG); Lancosamide (LCS); Levetiracetam (LEV); PER (Perampanel); Phenytoin (PHT); Rufinamide (RUF); Synaptic vesicle protein 2A (SV2A); Tetrahydrocannabinolic acid (THCA); Topiramate (TPM); Triple transgenic (3×Tg) mouse model; Valproic acid (VPA); Zonisamide (ZNS).

## Data Availability

No new data were created or analyzed in this study. Data sharing is not applicable to this article.

## References

[1] Scearce-Levie K., Sanchez P.E., Lewcock J.W. (2020). Leveraging preclinical models for the development of Alzheimer disease therapeutics. Nat. Rev. Drug Discov..

[2] Scheltens P., De Strooper B., Kivipelto M., Holstege H., Chételat G., Teunissen C.E., Cummings J., van der Flier W.M. (2021). Alzheimer’s disease. Lancet.

[3] Long J.M., Holtzman D.M. (2019). Alzheimer Disease: An Update on Pathobiology and Treatment Strategies. Cell.

[4] Leo A., Tallarico M., Sciaccaluga M., Citraro R., Costa C. (2022). Epilepsy and Alzheimer’s Disease: Current Concepts and Treatment Perspective on Two Closely Related Pathologies. Curr. Neuropharmacol..

[5] Sen A., Jette N., Husain M., Sander J.W. (2020). Epilepsy in older people. Lancet.

[6] Vossel K.A., Tartaglia M.C., Nygaard H.B., Zeman A.Z., Miller B.L. (2017). Epileptic activity in Alzheimer’s disease: Causes and clinical relevance. Lancet Neurol..

[7] Helmstaedter C., Witt J.A. (2017). Epilepsy and cognition—A bidirectional relationship?. Seizure.

[8] Asadollahi M., Atazadeh M., Noroozian M. (2019). Seizure in Alzheimer’s Disease: An Underestimated Phenomenon. Am. J. Alzheimer’s Dis. Other Demen..

[9] Yang F., Chen L., Yu Y., Xu T., Chen L., Yang W., Wu Q., Han Y. (2022). Alzheimer’s disease and epilepsy: An increasingly recognized comorbidity. Front. Aging Neurosci..

[10] Giorgi F.S., Saccaro L.F., Busceti C.L., Biagioni F., Fornai F. (2020). Epilepsy and Alzheimer’s Disease: Potential mechanisms for an association. Brain Res. Bull..

[11] Palop J.J., Mucke L. (2009). Epilepsy and cognitive impairments in alzheimer disease. Arch. Neurol..

[12] Sen A., Capelli V., Husain M. (2018). Cognition and dementia in older patients with epilepsy. Brain.

[13] Bero A.W., Yan P., Roh J.H., Cirrito J.R., Stewart F.R., Raichle M.E., Lee J.M., Holtzman D.M. (2011). Neuronal activity regulates the regional vulnerability to amyloid-β 2 deposition. Nat. Neurosci..

[14] Cirrito J.R., Yamada K.A., Finn M.B., Sloviter R.S., Bales K.R., May P.C., Schoepp D.D., Paul S.M., Mennerick S., Holtzman D.M. (2005). Synaptic activity regulates interstitial fluid amyloid-β levels in vivo. Neuron.

[15] Yamamoto K., Tanei Z.I., Hashimoto T., Wakabayashi T., Okuno H., Naka Y., Yizhar O., Fenno L.E., Fukayama M., Bito H. (2015). Chronic Optogenetic Activation Augments Aβ Pathology in a Mouse Model of Alzheimer Disease. Cell Rep..

[16] Romoli M., Sen A., Parnetti L., Calabresi P., Costa C. (2021). Amyloid-β: A potential link between epilepsy and cognitive decline. Nat. Rev. Neurol..

[17] Sciaccaluga M., Megaro A., Bellomo G., Ruffolo G., Romoli M., Palma E., Costa C. (2021). An unbalanced synaptic transmission: Cause or consequence of the amyloid oligomers neurotoxicity?. Int. J. Mol. Sci..

[18] Yang J.W., Czech T., Felizardo M., Baumgartner C., Lubec G. (2006). Aberrant expression of cytoskeleton proteins in hippocampus from patients with mesial temporal lobe epilepsy. Amino Acids.

[19] Choi J., Kim S.Y., Kim H., Lim B.C., Hwang H., Chae J.H., Kim K.J., Oh S., Kim E.Y., Shin J.S. (2020). Serum α-synuclein and IL-1β are increased and correlated with measures of disease severity in children with epilepsy: Potential prognostic biomarkers?. BMC Neurol..

[20] Li A., Choi Y.S., Dziema H., Cao R., Cho H.Y., Jung Y.J., Obrietan K. (2010). Proteomic profiling of the epileptic dentate gyrus. Brain Pathol..

[21] Hussein A.M., Eldosoky M., El-Shafey M., El-Mesery M., Ali A.N., Abbas K.M., Abulseoud O.A. (2019). Effects of metformin on apoptosis and α-synuclein in a rat model of pentylenetetrazole-induced epilepsy. Can. J. Physiol. Pharmacol..

[22] Dejakaisaya H., Kwan P., Jones N.C. (2021). Astrocyte and glutamate involvement in the pathogenesis of epilepsy in Alzheimer’s disease. Epilepsia.

[23] Langworth-Green C., Patel S., Jaunmuktane Z., Jabbari E., Morris H., Thom M., Lees A., Hardy J., Zandi M., Duff K. (2023). Chronic effects of inflammation on tauopathies. Lancet Neurol..

[24] Tang T., Li X., Yu E., Li M., Pan X. (2023). Identification of common core ion channel genes in epilepsy and Alzheimer’s disease. Ir. J. Med. Sci..

[25] Perversi F., Costa C., Labate A., Lattanzi S., Liguori C., Maschio M., Meletti S., Nobili L., Operto F.F., Romigi A. (2023). The broad-spectrum activity of perampanel: State of the art and future perspective of AMPA antagonism beyond epilepsy. Front. Neurol..

[26] Leo A., Giovannini G., Russo E., Meletti S. (2018). The role of AMPA receptors and their antagonists in status epilepticus. Epilepsia.

[27] Tallarico M., Leo A., Russo E., Citraro R., Palma E., De Sarro G. (2023). Seizure susceptibility to various convulsant stimuli in the BTBR mouse model of autism spectrum disorders. Front. Pharmacol..

[28] Costa C., Parnetti L., D’Amelio M., Tozzi A., Tantucci M., Romigi A., Siliquini S., Cavallucci V., Di Filippo M., Mazzocchetti P. (2016). Epilepsy, amyloid-β, and D1 dopamine receptors: A possible pathogenetic link?. Neurobiol. Aging.

[29] Palop J.J., Chin J., Roberson E.D., Wang J., Thwin M.T., Bien-Ly N., Yoo J., Ho K.O., Yu G.Q., Kreitzer A. (2007). Aberrant Excitatory Neuronal Activity and Compensatory Remodeling of Inhibitory Hippocampal Circuits in Mouse Models of Alzheimer’s Disease. Neuron.

[30] Bellingacci L., Tallarico M., Mancini A., Megaro A., De Caro C., Citraro R., De Sarro G., Tozzi A., Di Filippo M., Sciaccaluga M. (2023). Non-competitive AMPA glutamate receptors antagonism by perampanel as a strategy to counteract hippocampal hyper-excitability and cognitive deficits in cerebral amyloidosis. Neuropharmacology.

[31] Zott B., Konnerth A. (2023). Impairments of glutamatergic synaptic transmission in Alzheimer’s disease. Semin. Cell Dev. Biol..

[32] Beghi E., Beghi M. (2020). Epilepsy, antiepileptic drugs and dementia. Curr. Opin. Neurol..

[33] Lozano R., Fullman N., Mumford J.E., Knight M., Barthelemy C.M., Abbafati C., Abbastabar H., Abd-Allah F., Abdollahi M., Abedi A. (2020). Measuring universal health coverage based on an index of effective coverage of health services in 204 countries and territories, 1990–2019: A systematic analysis for the Global Burden of Disease Study 2019. Lancet.

[34] Müller L., Kirschstein T., Köhling R., Kuhla A., Teipel S. (2021). Neuronal Hyperexcitability in APPSWE/PS1dE9 Mouse Models of Alzheimer’s Disease. J. Alzheimer’s Dis..

[35] Altuna M., Olmedo-Saura G., Carmona-Iragui M., Fortea J. (2022). Mechanisms Involved in Epileptogenesis in Alzheimer’s Disease and Their Therapeutic Implications. Int. J. Mol. Sci..

[36] Powell G., Ziso B., Larner A.J. (2019). The overlap between epilepsy and Alzheimer’s disease and the consequences for treatment. Expert Rev. Neurother..

[37] Ziyatdinova S., Viswanathan J., Hiltunen M., Tanila H., Pitkänen A. (2015). Reduction of epileptiform activity by valproic acid in a mouse model of Alzheimer’s disease is not long-lasting after treatment discontinuation. Epilepsy Res..

[38] Ziyatdinova S., Gurevicius K., Kutchiashvili N., Bolkvadze T., Nissinen J., Tanila H., Pitkänen A. (2011). Spontaneous epileptiform discharges in a mouse model of Alzheimer’s disease are suppressed by antiepileptic drugs that block sodium channels. Epilepsy Res..

[39] Qing H., He G., Ly P.T.T., Fox C.J., Staufenbiel M., Cai F., Zhang Z., Wei S., Sun X., Chen C.H. (2008). Valproic acid inhibits aβ production, neuritic plaque formation, and behavioral deficits in alzheimer’s disease mouse models. J. Exp. Med..

[40] Grimes C.A., Jope R.S. (2001). The multifaceted roles of glycogen synthase kinase 3β in cellular signaling. Prog. Neurobiol..

[41] Mark R.J., Wesson Ashford J., Goodman Y., Mattson M.P. (1995). Anticonvulsants attenuate amyloid β-peptide neurotoxicity, Ca^2+^ deregulation, and cytoskeletal pathology. Neurobiol. Aging.

[42] Barker-Haliski M., White H.S. (2015). Glutamatergic mechanisms associated with seizures and epilepsy. Cold Spring Harb. Perspect. Med..

[43] Zhang H., Jiang X., Ma L., Wei W., Li Z., Chang S., Wen J., Sun J., Li H. (2022). Role of Aβ in Alzheimer’s-related synaptic dysfunction. Front. Cell Dev. Biol..

[44] Whitcomb D.J., Hogg E.L., Regan P., Piers T., Narayan P., Whitehead G., Winters B.L., Kim D.H., Kim E., St George-Hyslop P. (2015). Intracellular oligomeric amyloid-beta rapidly regulates GluA1 subunit of AMPA receptor in the hippocampus. Sci. Rep..

[45] Díaz-Alonso J., Nicoll R.A. (2021). AMPA receptor trafficking and LTP: Carboxy-termini, amino-termini and TARPs. Neuropharmacology.

[46] Nakajima M., Suda S., Sowa K., Sakamoto Y., Nito C., Nishiyama Y., Aoki J., Ueda M., Yokobori S., Yamada M. (2018). AMPA Receptor Antagonist Perampanel Ameliorates Post-Stroke Functional and Cognitive Impairments. Neuroscience.

[47] Zhang L., Wang L., Wang R., Gao Y., Che H., Pan Y., Fu P. (2017). Evaluating the effectiveness of GTM-1, rapamycin, and carbamazepine on autophagy and Alzheimer disease. Med. Sci. Monit..

[48] Nygaard H.B., Kaufman A.C., Sekine-Konno T., Huh L.L., Going H., Feldman S.J., Kostylev M.A., Strittmatter S.M. (2015). Brivaracetam, but not ethosuximide, reverses memory impairments in an Alzheimer’s disease mouse model. Alzheimer’s Res. Ther..

[49] Sanchez P.E., Zhu L., Verret L., Vossel K.A., Orr A.G., Cirrito J.R., Devidze N., Ho K., Yu G.Q., Palop J.J. (2012). Levetiracetam suppresses neuronal network dysfunction and reverses synaptic and cognitive deficits in an Alzheimer’s disease model. Proc. Natl. Acad. Sci. USA.

[50] Morris M., Sanchez P.E., Verret L., Beagle A.J., Guo W., Dubal D., Ranasinghe K.G., Koyama A., Ho K., Yu G.Q. (2015). Network dysfunction in α-synuclein transgenic mice and human Lewy body dementia. Ann. Clin. Transl. Neurol..

[51] He Y.X., Shen Q.Y., Tian J.H., Wu Q., Xue Q., Zhang G.P., Wei W., Liu Y.H. (2020). Zonisamide Ameliorates Cognitive Impairment by Inhibiting ER Stress in a Mouse Model of Type 2 Diabetes Mellitus. Front. Aging Neurosci..

[52] Strzelczyk A., Schubert-Bast S. (2022). Psychobehavioural and Cognitive Adverse Events of Anti-Seizure Medications for the Treatment of Developmental and Epileptic Encephalopathies. CNS Drugs.

[53] Chen B.H., Ahn J.H., Park J.H., Song M., Kim H., Lee T.K., Lee J.C., Kim Y.M., Hwang I.K., Kim D.W. (2018). Rufinamide, an antiepileptic drug, improves cognition and increases neurogenesis in the aged gerbil hippocampal dentate gyrus via increasing expressions of IGF-1, IGF-1R and p-CREB. Chem. Biol. Interact..

[54] Lai M.C., Wu S.N., Huang C.W. (2022). Rufinamide, a Triazole-Derived Antiepileptic Drug, Stimulates Ca^2+^-Activated K^+^ Currents While Inhibiting Voltage-Gated Na^+^ Currents. Int. J. Mol. Sci..

[55] Zhang M.Y., Zheng C.Y., Zou M.M., Zhu J.W., Zhang Y., Wang J., Liu C.F., Li Q.F., Xiao Z.C., Li S. (2014). Lamotrigine attenuates deficits in synaptic plasticity and accumulation of amyloid plaques in APP/PS1 transgenic mice. Neurobiol. Aging.

[56] Rizzello E., Pimpinella D., Pignataro A., Titta G., Merenda E., Saviana M., Porcheddu G.F., Paolantoni C., Malerba F., Giorgi C. (2023). Lamotrigine rescues neuronal alterations and prevents seizure-induced memory decline in an Alzheimer’s disease mouse model. Neurobiol. Dis..

[57] Fu X.X., Duan R., Wang S.Y., Zhang Q.Q., Wei B., Huang T., Gong P.Y., Yan E., Jiang T., Zhang Y.D. (2023). Lamotrigine protects against cognitive deficits, synapse and nerve cell damage, and hallmark neuropathologies in a mouse model of Alzheimer’s disease. Neural Regen. Res..

[58] Bang S.R., Ambavade S.D., Jagdale P.G., Adkar P.P., Waghmare A.B., Ambavade P.D. (2015). Lacosamide reduces HDAC levels in the brain and improves memory: Potential for treatment of Alzheimer’s disease. Pharmacol. Biochem. Behav..

[59] Ibrahim F.S., Abdel-Aziz L.F., Elbakly W.M., El Gayar N.H. (2021). Neuroprotective effect of lacosamide on cognitive dysfunction in Streptozotocin induced Alzheimer disease. QJM Int. J. Med..

[60] Alavi M.S., Fanoudi S., Hosseini M., Sadeghnia H.R. (2022). Beneficial effects of levetiracetam in streptozotocin-induced rat model of Alzheimer’s disease. Metab. Brain Dis..

[61] Fu C.H., Iascone D.M., Petrof I., Hazra A., Zhang X., Pyfer M.S., Tosi U., Corbett B.F., Cai J., Lee J. (2019). Early Seizure Activity Accelerates Depletion of Hippocampal Neural Stem Cells and Impairs Spatial Discrimination in an Alzheimer’s Disease Model. Cell Rep..

[62] Silva J.C., Shen Y., Chan J., Kwan P., Jones N.C. (2022). Anti-epileptogenic effects of synaptic vesicle protein 2A modulation in a mouse model of Alzheimer’s disease. Epilepsy Res..

[63] Shi J.Q., Wang B.R., Tian Y.Y., Xu J., Gao L., Zhao S.L., Jiang T., Xie H.G., Zhang Y.D. (2013). Antiepileptics Topiramate and Levetiracetam Alleviate Behavioral Deficits and Reduce Neuropathology in APPswe/PS1dE9 Transgenic Mice. CNS Neurosci. Ther..

[64] Cheng X.L., Li M.K. (2014). Effect of topiramate on apoptosis-related protein expression of hippocampus in model rats with Alzheimers Disease. Eur. Rev. Med. Pharmacol. Sci..

[65] Owona B.A., Zug C., Schluesener H.J., Zhang Z.Y. (2019). Amelioration of Behavioral Impairments and Neuropathology by Antiepileptic Drug Topiramate in a Transgenic Alzheimer’s Disease Model Mice, APP/PS1. Int. J. Mol. Sci..

[66] Loring D.W., Williamson D.J., Meador K.J., Wiegand F., Hulihan J. (2011). Topiramate dose effects on cognition: A randomized double-blind study. Neurology.

[67] Meador K.J., Loring D.W., Hulihan J.F., Kamin M., Karim R. (2003). Differential cognitive and behavioral effects of topiramate and valproate. Neurology.

[68] Besag F.M.C., Vasey M.J. (2021). Neurocognitive Effects of Antiseizure Medications in Children and Adolescents with Epilepsy. Paediatr. Drugs.

[69] Ryan M. (2020). Cannabidiol in epilepsy: The indications and beyond. Ment. Health Clin..

[70] Leo A., Russo E., Elia M. (2016). Cannabidiol and epilepsy: Rationale and therapeutic potential. Pharmacol. Res..

[71] O’Sullivan S.E., Jensen S.S., Nikolajsen G.N., Bruun H.Z., Bhuller R., Hoeng J. (2023). The therapeutic potential of purified cannabidiol. J. Cannabis Res..

[72] Xiong Y., Lim C.S. (2021). Understanding the modulatory effects of cannabidiol on alzheimer’s disease. Brain Sci..

[73] Esposito G., Scuderi C., Savani C., Steardo L., De Filippis D., Cottone P., Iuvone T., Cuomo V., Steardo L. (2007). Cannabidiol in vivo blunts β-amyloid induced neuroinflammation by suppressing IL-1β and iNOS expression. Br. J. Pharmacol..

[74] Martín-Moreno A.M., Reigada D., Ramírez B.G., Mechoulam R., Innamorato N., Cuadrado A., De Ceballos M.L. (2011). Cannabidiol and other cannabinoids reduce microglial activation in vitro and in vivo: Relevance to alzheimer’s disease. Mol. Pharmacol..

[75] Cheng D., Low J.K., Logge W., Garner B., Karl T. (2014). Chronic cannabidiol treatment improves social and object recognition in double transgenic APPswe/PS1ΔE9 mice. Psychopharmacology.

[76] Cheng D., Spiro A.S., Jenner A.M., Garner B., Karl T. (2014). Long-term cannabidiol treatment prevents the development of social recognition memory deficits in alzheimer’s disease transgenic mice. J. Alzheimer’s Dis..

[77] Watt G., Shang K., Zieba J., Olaya J., Li H., Garner B., Karl T. (2020). Chronic Treatment with 50 mg/kg Cannabidiol Improves Cognition and Moderately Reduces Aβ40 Levels in 12-Month-Old Male AβPPswe/PS1ΔE9 Transgenic Mice. J. Alzheimer’s Dis..

[78] Kim J., Choi P., Park Y.T., Kim T., Ham J., Kim J.C. (2023). The Cannabinoids, CBDA and THCA, Rescue Memory Deficits and Reduce Amyloid-Beta and Tau Pathology in an Alzheimer’s Disease-like Mouse Model. Int. J. Mol. Sci..

[79] Vande Vyver M., Barker-Haliski M., Aourz N., Nagels G., Bjerke M., Engelborghs S., De Bundel D., Smolders I. (2022). Higher susceptibility to 6 Hz corneal kindling and lower responsiveness to antiseizure drugs in mouse models of Alzheimer’s disease. Epilepsia.

[80] Reardon S. (2018). Frustrated Alzheimer’s researchers seek better lab mice. Nature.

[81] LaFerla F.M., Green K.N. (2012). Animal models of Alzheimer disease. Cold Spring Harb. Perspect. Med..

[82] Foidl B., Humpel C. (2020). Can mouse models mimic sporadic Alzheimer’s disease?. Neural Regen. Res..

[83] Vitek M.P., Araujo J.A., Fosse M., Greenberg B.D., Howell G.R., Rizzo S.J.S., Seyfried N.T., Tenner A.J., Territo P.R., Windisch M. (2020). Translational animal models for Alzheimer’s disease: An Alzheimer’s Association Business Consortium Think Tank. Alzheimer’s Dement. Transl. Res. Clin. Interv..

[84] Lehmann L., Lo A., Knox K.M., Barker-Haliski M. (2021). Alzheimer’s Disease and Epilepsy: A Perspective on the Opportunities for Overlapping Therapeutic Innovation. Neurochem. Res..

[85] Giorgi F.S., Guida M., Vergallo A., Bonuccelli U., Zaccara G. (2017). Treatment of epilepsy in patients with Alzheimer’s disease. Expert Rev. Neurother..

[86] Kaur U., Chauhan I., Gambhir I.S., Chakrabarti S.S. (2019). Antiepileptic drug therapy in the elderly: A clinical pharmacological review. Acta Neurol. Belg..

[87] Devinsky O., Vezzani A., O’Brien T.J., Jette N., Scheffer I.E., De Curtis M., Perucca P. (2018). Epilepsy. Nat. Rev. Dis. Primers.

[88] Motika P.V., Spencer D.C. (2016). Treatment of Epilepsy in the Elderly. Curr. Neurol. Neurosci. Rep..

[89] Eyer F., Felgenhauer N., Gempel K., Steimer W., Gerbitz K.D., Zilker T. (2005). Acute valproate poisoning: Pharmacokinetics, alteration in fatty acid metabolism, and changes during therapy. J. Clin. Psychopharmacol..

[90] Rogawski M.A., Löscher W., Rho J.M. (2016). Mechanisms of action of Antiseizure Drugs and the Ketogenic diet. Cold Spring Harb. Perspect. Med..

[91] Kanner A.M., Helmstaedter C., Sadat-Hossieny Z., Meador K. (2020). Cognitive disorders in epilepsy I: Clinical experience, real-world evidence and recommendations. Seizure.

[92] Tallarico M., Pisano M., Leo A., Russo E., Citraro R., De Sarro G. (2023). Antidepressants drugs for seizures and epilepsy: Where do we stand?. Curr. Neuropharmacol..

[93] Perucca E., Brodie M.J., Kwan P., Tomson T. (2020). 30 Years of Second-Generation Antiseizure Medications: Impact and Future Perspectives. Lancet Neurol..

[94] Cretin B. (2021). Treatment of Seizures in Older Patients with Dementia. Drugs Aging.

[95] Rowan A.J., Ramsay R.E., Collins J.F., Pryor F., Boardman K.D., Uthman B.M., Spitz M., Frederick T., Towne A., Carter G.S. (2005). New onset geriatric epilepsy: A randomized study of gabapentin, lamotrigine, and carbamazepine. Neurology.

[96] Saetre E., Perucca E., Isojärvi J., Gjerstad L., Babic T., Hodoba D., Vrca A., Lusic I., Kälviäinen R., Keränen T. (2007). An international multicenter randomized double-blind controlled trial of lamotrigine and sustained-release carbamazepine in the treatment of newly diagnosed epilepsy in the elderly. Epilepsia.

[97] Brodie M.J., Overstall P.W., Giorgi L. (1999). Multicentre, double-blind, randomised comparison between lamotrigine and carbamazepine in elderly patients with newly diagnosed epilepsy. Epilepsy Res..

[98] Arif H., Buchsbaum R., Pierro J., Whalen M., Sims J., Resor S.R., Bazil C.W., Hirsch L.J. (2010). Comparative effectiveness of 10 antiepileptic drugs in older adults with epilepsy. Arch. Neurol..

[99] Tariot P.N., Schneider L.S., Cummings J., Thomas R.G., Raman R., Jakimovich L.J., Loy R., Bartocci B., Fleisher A., Ismail M.S. (2011). Chronic divalproex sodium to attenuate agitation and clinical progression of Alzheimer disease. Arch. Gen. Psychiatry.

[100] Profenno L., Jakimovich L., Holt C., Porsteinsson A., Tariot P. (2005). A Randomized, Double-Blind, Placebo-Controlled Pilot Trial of Safety and Tolerability of Two Doses of Divalproex Sodium in Outpatients with Probable Alzheimers Disease. Curr. Alzheimer Res..

[101] Fleisher A.S., Truran D., Mai J.T., Langbaum J.B.S., Aisen P.S., Cummings J.L., Jack C.R., Weiner M.W., Thomas R.G., Schneider L.S. (2011). Chronic divalproex sodium use and brain atrophy in Alzheimer disease. Neurology.

[102] Callisto S.P., Illamola S.M., Birnbaum A.K., Barkley C.M., Bathena S.P.R., Leppik I.E., Marino S.E. (2020). Severity of Topiramate-Related Working Memory Impairment Is Modulated by Plasma Concentration and Working Memory Capacity. J. Clin. Pharmacol..

[103] Eun S.H., Kim H.D., Eun B.L., Lee I.K., Chung H.J., Kim J.S., Kang H.C., Lee Y.M., Suh E.S., Kim D.W. (2011). Comparative trial of low- and high-dose zonisamide as monotherapy for childhood epilepsy. Seizure.

[104] Mendez M.F., Lim G.T.H. (2003). Seizures in elderly patients with dementia: Epidemiology and management. Drugs Aging.

[105] Cumbo E., Ligori L.D. (2010). Levetiracetam, lamotrigine, and phenobarbital in patients with epileptic seizures and Alzheimer’s disease. Epilepsy Behav..

[106] Carter M.D., Weaver D.F., Joudrey H.R., Carter A.O., Rockwood K. (2007). Epilepsy and antiepileptic drug use in elderly people as risk factors for dementia. J. Neurol. Sci..

[107] Rao S.C., Dove G., Cascino G.D., Petersen R.C. (2009). Recurrent seizures in patients with dementia: Frequency, seizure types, and treatment outcome. Epilepsy Behav..

[108] Liu J., Wang L.N. (2021). Treatment of epilepsy for people with Alzheimer’s disease. Cochrane Database Syst. Rev..

[109] Vossel K., Ranasinghe K.G., Beagle A.J., La A., Ah Pook K., Castro M., Mizuiri D., Honma S.M., Venkateswaran N., Koestler M. (2021). Effect of Levetiracetam on Cognition in Patients with Alzheimer Disease with and without Epileptiform Activity: A Randomized Clinical Trial. JAMA Neurol..

[110] Hautecloque-Raysz G., Sellal F., Bousiges O., Phillipi N., Blanc F., Cretin B. (2023). Epileptic Prodromal Alzheimer’s Disease Treated with Antiseizure Medications: Medium-Term Outcome of Seizures and Cognition. J. Alzheimer’s Dis..

[111] del Pozo A., Lehmann L., Knox K.M., Barker-Haliski M. (2022). Can Old Animals Reveal New Targets? The Aging and Degenerating Brain as a New Precision Medicine Opportunity for Epilepsy. Front. Neurol..

